# Exploring dysphagia workload, aetiologies and preferred intervention strategies: Pakistani speech-language pathologists’ perspective

**DOI:** 10.4102/sajcd.v73i1.1181

**Published:** 2026-09-16

**Authors:** Arshad Mahmood Naz, Nazia Mumtaz, Ghulam Saqulain

**Affiliations:** 1 Department of Speech Language Pathology, Faculty of Rehab and Allied Health Sciences, Riphah International University, Lahore, Pakistan; 2 Department of Speech Language Pathology, Mayo Hospital, Lahore, Pakistan; 3 Department of Otorhinolaryngology, Riphah International Hospital, Riphah International University, Islamabad, Pakistan

**Keywords:** dysphagia, aetiology, intervention, speech-language pathologists, swallowing

## Abstract

**Background:**

Clinicians may encounter cases with swallowing difficulties and aspiration risks. The intervention depends upon multiple factors. A professional decides on an appropriate intervention protocol. Therefore, exploring clinicians’ workload and intervention strategies is essential.

**Objectives:**

To explore the speech-language pathologists’ (SLPs) perspective regarding workload, aetiologies, and management, including their most preferred intervention approaches to manage clients with dysphagia.

**Method:**

This qualitative exploratory research was conducted at Riphah International University from January 2023 to December 2023, employing a sample of 22 SLPs aged 25–55 years, either gender, with at least a master’s qualification and 2 years of experience. The study utilised detailed interviews aided with an interview guide, duly validated and piloted beforehand and followed by thematic analysis.

**Results:**

The study revealed a heavy workload being catered by SLPs, with six themes: (1) acquired aetiological conditions with stroke, traumatic brain injury being commonest subthemes, (2) current trend of intervention with a subtheme need for electrical stimulation, (3) traditional strategies, (4) multidisciplinary team referrals, (5) need of awareness campaigns and (6) limitation of access to services.

**Conclusion:**

The study divulged that the services for individuals with dysphagia are concentrated primarily in major urban centres. The service delivery models are not standardised, rely on traditional support, and need to be made robust and evidence-based. There is a dearth of technological reliance; thus, standardised tools and equipment are strongly mandated. There is a higher workload of SLPs; a multidisciplinary team approach and awareness campaigns are essential.

**Contribution:**

This study has contributed to the growing body of literature in terms of SLPs’ perspective regarding workload, aetiologies, and management, including the most preferred intervention approaches to manage clients with dysphagia. This study is of significance as it will help with prevention, management, and information dissemination to the subject population, as well as for research purposes.

## Introduction

Swallowing, a process dedicated to moving the food bolus to the stomach, entails both mechanical and neurological support, which itself is both a sophisticated and synchronic activity. Although swallowing may appear deceptively simple, it is in fact a highly intricate neuromuscular process that relies on precise coordination among cortical, subcortical, and brainstem structures. Cortical regions initiate and regulate the voluntary oral phase, while subcortical circuits fine-tune motor control. The brainstem central pattern generator integrates sensory input with cranial nerve activity (V, VII, IX, X, XII) to orchestrate rhythmic, involuntary movements of the pharynx and oesophagus. This seamless interplay ensures airway protection, efficient bolus transit, and safe progression from voluntary to reflexive phases. Any disruption in these interconnected neural pathways can compromise swallowing efficiency and lead to dysphagia (Walton & Silva, 2024). Impairments arising from neurological, physiological, or anatomical factors can result in atypical swallowing patterns. The consequent aspiration may lead to life-threatening conditions such as pneumonia or manifest as struggling behaviours that render the swallowing process more laborious and fatiguing (Panara & Padalia, 2020). Dysphagia is a highly prevalent condition ranging from 2% to 16% among the general population and 40% in hospitalised cases, with a higher risk in the older population (Baijens et al., 2016). In the Indo-Pak subcontinent, recent studies highlight a significant prevalence of dysphagia among older adults, with community-based surveys in India having reported rates ranging up to 36.6%. Although direct prevalence data from Pakistan remain limited, the presence of similar risk factors – such as ageing, stroke, and neurological disease – suggests that comparable rates are likely within Pakistani populations (Dodderi et al., 2024). A very high prevalence of 38% to 53% in post-stroke cases has been reported in Pakistan (Akhtar et al., 2023). Dysphagia, commonly known as swallowing difficulty, is a condition in which the safe passage of food from the oral cavity to the stomach is interrupted. Dysphagia can result from neurological disorders like stroke, multiple sclerosis, Parkinson’s and neurological trauma; muscle dysfunction like oesophageal spasm, dystrophy or achalasia; or structural abnormalities in the throat and oesophagus including neoplasms, stricture, diverticulum etc (Wei et al., 2023). Literature revealed that among the four dysphagia stages, including oral preparatory, oral, pharyngeal, and oesophageal phases, the cardiovascular accidents (CVAs) frequently affect the first three stages by reducing sensations, conscious control of mastication, eliciting swallowing reflex, et cetera. Disorders or lesions affecting these cortical areas may result in abnormal lip closure, incoordination, delays, and often a complete absence of gag reflex (Abubakar & Jamoh, 2017). Among the four identified stages of swallowing, the oropharyngeal phase is more frequently affected and related to aspiration, severe dietary, nutritional, and pulmonary difficulties. Cases with dysphagia often remain neglected (Clavé & Shaker, 2015). An atypical swallowing pattern may result in aspiration and can lead to life-threatening conditions like pneumonia or may result in struggling behaviour, making the process more laborious and tiring (Panara & Padalia, 2020). Dysphagia is also associated with a decrease in quality of life, dehydration, malnutrition, and social isolation, necessitating dysphagia screening (Andrade et al., 2018). Literature revealed that among the speech-language pathologists’ (SLPs’) perceptions regarding dysphagia, they highlighted it as challenging with complications in the acute phase following stroke (Minchell et al., 2022). As dysphagia can lead to life-threatening situations, there is a need for further study (Dziewas et al., 2021). Although SLPs’ work in dysphagia in South American countries, including Brazil, has been reported as impressive (Mendes et al., 2022), recent surveys from both Pakistan and India reveal that many SLPs lack sufficient knowledge, training, and standardised practices in the management of dysphagia, hence dysphagia cases form a very small percentage of their overall workload with 79% having less than 5 years of dysphagia-related practice. This underscores the urgent need for professional development initiatives (Akhtar et al., 2023; Rangarathnam & Desai, 2020). Another Indian study revealed that SLPs do not receive enough chances to work in dysphagia multidisciplinary teams (Devan & Mk, 2023), although there are several benefits of working in multidisciplinary teams (Brammer & Vicari, 2020). A previous study reveals that SLPs in Pakistan demonstrate limited and variable knowledge of dysphagia aetiologies, often recognising neurological causes like stroke and Parkinson’s (Akhtar et al., 2023). Also, according to Haider and Zubair (2022), in Pakistan, the SLPs’ most preferred intervention approaches for dysphagia management are primarily behavioural and compensatory techniques, while advanced instrumental methods are marred by limited use. Dysphagia is a highly prevalent condition with significant health implications, particularly in post-stroke populations where rates in Pakistan have been reported as high as 38% – 53% (Akhtar et al., 2023). Its management is critical, given the life-threatening complications such as aspiration pneumonia, malnutrition, and dehydration (Dziewas et al., 2021). Effective care requires specialised knowledge, skills, and clearly defined roles for SLPs, yet evidence from the Indo-Pak region highlights persistent limitations in current practice. Studies reveal that many SLPs lack adequate training, standardised protocols, and opportunities to work within multidisciplinary teams, all of which are essential for optimal dysphagia management (Devan & Mk, 2023). These gaps underscore the pressing need to investigate SLPs’ perspectives regarding their workload, understanding of dysphagia aetiologies, and preferred management approaches. Such exploration will help identify deficiencies in knowledge, training, and practice, thereby guiding the development of targeted professional development initiatives and standardised guidelines. Strengthening dysphagia care in this way is vital to improving patient outcomes in Pakistan and similar contexts. Moreover, this study holds significant importance as it will contribute to prevention, enhance management strategies, and support information dissemination to both clinical populations and the research community.

## Research methods and design

The current qualitative exploratory research was conducted at Riphah International University over a period of 12 months, from 01 January 2023 to 31 December 2023. The study was conducted after obtaining approval from the Research Ethics Committee of Riphah International University, Lahore.

### Sample and sampling technique

A purposive sample of 22 SLPs was recruited from public and private hospitals offering services to clients with dysphagia, with data collection continuing until saturation was achieved – no substantively new themes emerged after the 20th interview, and two additional interviews confirmed saturation in line with established qualitative adequacy standards. The sample included SLPs aged between 25 years and 55 years, either gender, with a minimum qualification of a master’s degree in SLP and 2 years of experience in dysphagia management. Speech-language pathologists working in administrative positions were excluded from the study.

### Instrument

Considering the study’s objective, in-depth interviews with open-ended questions were chosen in line with the qualitative paradigm of the research, using prompts or probes to elicit responses. For this purpose, the first phase was to develop an interview guide, establish content validity, and pilot the process. The interview guide was developed based on gaps mentioned in the literature about the perceptions and confidence of the professionals on intervention options. The group of participants for the interview was selected, and questions were developed based on the themes emerging from the literature review. The key themes that emerged from the literature included professional skills, technological support, and workload, which can affect the efficacy of treatment in such cases. Once developed, the guide was externally reviewed by professionals and experts in the field. Revision of weaker statements and ambiguities was improved to make the tool more robust and user-friendly. Likewise, some questions considered irrelevant were omitted. The process also entailed revising prompts and probes to increase the reliability of the tool, as recommended by Wang et al. (2024). It was ensured that the questions were easy to understand and well-sequenced. The tool was duly validated by the research supervisor and professionals working in public sector hospitals and piloted on three SLPs as per the inclusion criteria. Based on expert reviewer feedback, some items were rephrased and/or excluded before the final draft.

### Procedure

The data collection procedure was followed with well-timed appointments and consent, followed by an in-depth interview using the interview guide. Interviews were recorded using a digital voice recorder while queries were put forward to the participants to gather their responses. This helped the researcher with theme extraction, giving participants time to respond uninhibitedly, while probes were used as needed. The current study employed a thematic analysis framework following Braun and Clarke’s (2006) six-phase process, which emphasises systematic coding and theme development instead of adherence to a specific philosophical tradition such as phenomenology or grounded theory. We followed the six-phase thematic analysis framework as described below:

#### Phase 1 – Familiarisation with data

The recorded data were subjected to analysis, with the digital recordings transcribed verbatim.

#### Phase 2 – Generating initial codes

Following familiarisation, an exploration of initial broad codes was done, followed by organisation of these experiences and views.

#### Phase 3 – Searching for themes

This exercise helped in identifying the key themes. Subthemes were derived from the main theme and used to generate responses from the SLPs.

#### Phase 4 – Reviewing themes

Themes are checked against the data, refined, merged, or discarded.

#### Phase 5 – Defining and naming themes

New themes emerging from the data that did not fit the agreed codes were assigned new ones. Clear definitions and labels are given to themes, ensuring they capture the essence of the data.

#### Phase 6 – Producing the report

Final fine-tuning was done to develop consistent and rational themes and subthemes.

To ensure trustworthiness, the authors adhered to Lincoln and Guba’s (1985) criteria of credibility, transferability, dependability, and confirmability. Credibility was supported through prolonged engagement and expert review of the interview guide; transferability through thick description of the sample and setting; dependability through a documented audit trail of the coding process; and confirmability through supervisor oversight of theme development. Reflexivity was maintained throughout the research process, with the researcher critically reflecting on personal assumptions, positionality, and potential influence on data interpretation.

### Ethical considerations

Ethical clearance to conduct this study was obtained from the Riphah International University Research Ethics Committee (No. F20C14P17005). The study was conducted in accordance with relevant guidelines and/or regulations. Informed consent was obtained from all the participants involved in the study.

## Results

The results of this study indicate that the majority of the respondent SLPs were females (54%) and held a master’s degree in speech-language pathology. The mean age of the respondents was 38.45 ± 9.29 years, with a mean experience of 12.95 ± 7.73 years. The engagement hours of practice per day worked out as 8 h/day, while the average number of dysphagic patients seeking support was 7 (Table 1).

**TABLE 1:** Demographic characteristics of the sample (*N* = 22).

Variable	Group	*n*	%	Mean ± s.d.	Range
Age (years)	-	-	-	38.45 ± 9.30	27–53
Experience (years)	-	-	-	12.95 ± 7.74	3–30
Engagement/day (hours)	-	-	-	7.95 ± 1.31	6–10
Dysphagia patients/day	-	-	-	6.73 ± 4.15	1–15
Gender	Male	10	45.45	-	-
	Female	12	54.55	-	-
Qualification	PhD	2	9.09	-	-
	MS	20	90.91	-	-
Institution services	Public	6	27.30	-	-
	Private	12	54.50	-	-
	Both	4	18.20	-	-
Client age group	Children	4	18.20	-	-
	Adults	4	18.20	-	-
	Both	14	63.60	-	-

Note: Percentages may not total 100% due to rounding.

s.d., standard deviation; PhD, Doctor of Philosophy; MS, Master of Science.

Table 1 shows that 63.6% of SLPs served both children and adults, while 18.2% served children or adults alone. Traditional, non-invasive strategies dominated dysphagia intervention. Analysis suggests that SLPs’ dysphagia practice is shaped by three different focuses that include finding issues with the nervous system, navigating the evidence through formal and informal means, and negotiating the barriers imposed by the system, such as access and the non-availability of technological support. The following paragraph will reflect upon the qualitative findings that have emerged from Table 1. As a primary finding, the participants constructed dysphagia primarily as a problem of some neurological or bodily effect. When describing their caseloads, SLPs reported acquired neurological conditions – stroke, Traumatic Brain Injury (TBI), brain tumours, Parkinsonism, dementia, and Alzheimer’s and so on, with oropharyngeal dysphagia presentations. Secondly, intervention choices revealed a mismatch between standard options and available resources. While SLPs named electrical stimulation as the ‘current international trend’, their daily practice relied on what they called ‘traditional strategies’: postural management, manoeuvres, counselling, nutritional management, endoscopic intervention, and bedside swallow evaluations. Thirdly, participants described their work as necessarily collective but isolated in reality. Referrals to multidisciplinary teams were valued in principle; however, access to them was questioned. SLPs recommended awareness campaigns as a possible solution, targeting both professional training and family or caregiver education. Thus, ‘limitation of access’ was not merely a logistical barrier; it functioned as a rationing mechanism that SLPs navigated daily.

### Theme 1: Aetiologies and types

**Subtheme 1.1: Acquired conditions:** Clinical observations indicate that dysphagia is primarily secondary to neurogenic and non-modifiable medical factors. Practitioners frequently identify stroke as the most prevalent cause, followed by a range of neurological disorders (Table 2):

- **Participant perspectives:** The clinical landscape is dominated by ‘CVA [*Cerebrovascular Accident*], TBI [*Traumatic Brain Injury*], and other neurogenic disorders’ (P11–L5). Practitioners noted that the geriatric population frequently presents with ‘Parkinson, motor neurons disease’ (P9–L3), as well as dementia, Alzheimer’s, and brain tumours (P5–L1, P7–L2). It was emphasised that these aetiologies are ‘totally dependent upon medical factors and not modifiable’ (P2–L3).

**TABLE 2:** Themes and sub-themes.

Theme	Subtheme	Key findings and participant illustrative quotes
1. Aetiologies and types	1.1 Acquired conditions	Dysphagia is primarily secondary to neurogenic and non-modifiable factors. The clinical data are linked mostly to conditions like ‘CVA, TBI, and other neurogenic disorders’ (P11, L5). Common aetiologies include stroke, Parkinson’s, and MND (P4, L1).
2. Intervention strategies	2.1 Current trends	There is an emerging interest in ‘electrical stimulation’ (P1-5, L17); however, some clinicians have ‘not yet used’ it despite being aware of it (P6, L33).
	2.2 Traditional strategies	Standard care includes ‘postural management, swallowing manoeuvres, and diet modification’ (P2, L23). Decisions often involve ‘NG tube or PEG tube’ placement based on severity (P7, L18).
3. Referrals and teams	3.1 Multidisciplinary collaboration	Clinical pathways are established for ‘every patient presenting with stroke’ (P6, L32). Teams involve a liaison with other professionals for review of ‘CT scans, MRI, and labs’ (P5, L29).
4. Training and awareness	4.1 Professional and caregiver education	There is a critical need for ‘refresher courses for SLPs’ (P10, L81). Families require training because ‘they play a very important role’ in daily feeding and aftercare (P2, L65; P8, L48).
5. Rehabilitation services	5.1 Access limitations	A significant ‘dearth of professionals’ exists in rural or far-flung areas (P1-5, L20).
	5.2 Online modalities	Opinions on tele-rehabilitation vary; some argue ‘assessment should not be online’ (P2, L69), while others found it ‘helpful’ during the pandemic (P9, L71).

CVA, cerebrovascular accident; CT, computed tomography; MND, motor neuron disease; MRI, magnetic resonance imaging; NG, nasogastric; PEG, percutaneous endoscopic gastrostomy; TBI, traumatic brain injury; SLPs, speech-language pathologists.

### Theme 2: Intervention strategies

#### Subtheme 2.1: Current trends

There is an emerging interest in technological adjuncts to therapy, specifically electrical stimulation:

- **Participant perspectives:** While some clinicians are ‘considering using’ electrical stimulation as an option (P1–5, L17–20), others have ‘read about its usefulness’ but have not yet integrated it into their clinical practice (P6, L33).

#### Subtheme 2.2: Traditional strategies

Standard care remains rooted in compensatory and rehabilitative exercises, diet modification, and structural management:

- **Participant perspectives:** The foundational approach involves ‘postural management, guiding about swallowing manoeuvres, and diet modification’ (P2–L23). Initial critical decisions often involve nutritional access, specifically ‘whether to put the patient on an NG tube or a PEG tube’ (P7–L18). Advanced cases may require a referral for ‘endoscopic intervention to ENT specialists’ (P12–L11).

### Theme 3: Referrals and teams

#### Subtheme: 3.1: Multidisciplinary collaboration

**Interprofessional collaboration.** Effective management of dysphagia requires a robust clinical pathway involving neurologists, paediatricians, and rehabilitation specialists:

- **Participant perspectives:** Formalised ‘clinical pathways for referral’ exist for stroke patients (P6–L32). Collaboration is often intensive, including ‘weekly meetings with neuro physicians to discuss CT scans, MRI, and labs’ (P5–L29). Referrals typically flow from ‘paediatrics, rehabilitation medicine, neuro-physicians, and psychiatry’ (P3–L40), though an ‘ideal referral system’ is contingent upon the presence of a dedicated rehabilitation department (P4–L34).

### Theme 4: Training and awareness campaigns

#### Subtheme 4.1: Professional and caregiver education

There is a critical need for both specialised training for SLPs and education for family members:

- **Participant perspectives:** Clinicians agree that ‘trainings or refresher courses for SLPs are very important’ (P10–L81). Simultaneously, caregivers are viewed as essential partners because ‘aftercare by family members’ is constant (P8–L48). A lack of awareness often leads to ‘negligence and late referral leading to complications’ (P6–L73), making public awareness programmes vital.

### Theme 5: Rehabilitation services

#### Subtheme 5.1: Access limitations

Access to services is geographically skewed, with a significant ‘dearth of professionals’ in rural or ‘far-flung’ areas (P1–5, L20–25).

#### Subtheme 5.2: Online modalities

The efficacy of tele-rehabilitation remains a point of professional debate, specifically regarding assessment versus management:

- **Participant perspectives:** Some practitioners argue that ‘assessment should not be online’ because assessing muscle strength remotely is not feasible (P2–L69). However, others suggest that ‘if there is no other option available, then no harm in providing assessment online’ (P11–L81). Experience during the pandemic showed that online consultation was ‘helpful’ when caregivers assisted in applying techniques (P9–L71).

## Discussion

The present study is situated within the Pakistani context, where allied health professions such as speech-language pathology remain in their formative stages. While nursing has long been established, disciplines including occupational therapy, speech-language pathology, and vocational therapy are only beginning to gain recognition within educational and clinical faculties. Against this backdrop, the study sought to identify key factors contributing to dysphagia-like symptoms, particularly within the sociocultural realities of Pakistan. Structural challenges such as poverty, inadequate health and safety measures, political extremism, and volatile border conditions were anticipated to play a role in the neurological conditions that predispose individuals to dysphagia. At the same time, the study explored the intervention options currently available to dysphagic clients, recognising that the profession’s emerging status necessitates a clearer understanding of both workload distribution and preferred management approaches among clinicians. By situating these findings within the broader discourse on healthcare development, the study underscores the urgent need to establish foundational knowledge about professional practice patterns and intervention preferences in Pakistan, thereby contributing to the global conversation on dysphagia care in resource-constrained settings. This discussion will explore how the findings of the present study align with, extend, or diverge from existing literature. In the following sections, key results are examined in relation to prior evidence, highlighting areas of convergence that strengthen the credibility of the study as well as points of departure that invite further reflection. By situating the findings within established scholarship, the discussion aims to demonstrate both the continuity of evidence and the unique contributions this research makes to the broader understanding of dysphagia management. In terms of aetiologies, cerebrovascular accidents (CVA), TBI, brain tumours, and other neurological disorders, including Parkinson’s disease, were identified as leading causes of dysphagia in Pakistan. This finding is consistent with the earlier study conducted by Martino et al. (2005). Dysphagia has also been reported as an important non-motor feature of Parkinson’s disease (Kwon & Lee, 2019). Because dysphagia can arise from multiple aetiologies that are often difficult to predict and ascertain, neurological considerations remain the primary source of referrals made by professionals, with approximately 37% – 55% of cases resulting from stroke. Similarly, Menon (2022) reported that neurological causes, followed by pathological causes such as head and neck tumours, are common aetiologies, a finding echoed in the current study where one respondent stated: ‘It can be due to stroke or due to any kind of brain tumour’. Stroke has consistently been identified in the literature as a leading contributor to dysphagia-like symptoms, with reduced laryngeal elevation and delayed pharyngeal reflex among the common mechanisms that can also result in aspiration (Zhang et al., 2016). This aligns with the present study, where a respondent noted: ‘We usually see post-stroke dysphagia patients presenting with laryngeal dysphagia’. In addition, TBI cases were reported, with another respondent stating: ‘Mainly referrals are stroke patients of elderly ages and occasional accidental cases are referred to us’. Literature further supports this, revealing dysphagia in up to 93% of TBI cases (Howle et al., 2014). Literature reports dysphagia prevalence rates of 52% for tumours involving the pharynx, 28% for laryngeal tumours, and 21% for oral tumours (Cristofaro et al., 2021). However, the current study identified brain tumours as one of the leading causes. This difference may be explained by referral patterns, as patients with laryngopharyngeal and oral tumours typically present to otolaryngologists rather than to SLPs, thereby influencing the distribution of cases observed in this study. In the current study, one respondent observed: ‘We have a geriatric population with Parkinson’s, motor neuron disease’. Ageing itself, along with skeletal fragility, reduced daily life activities, and chronic illness, has been identified as a major risk factor for dysphagia (Dellis et al., 2018). Similar associations between age and swallowing difficulties have been consistently reported in the literature. For example, Dellis et al. (2018) reported a 15% prevalence among individuals aged 65 years and older. Age-related changes such as reduced muscle mass, decreased tissue elasticity, and diminished overall strength have been highlighted as contributing factors to dysphagia-like symptoms (Cichero, 2018). Moreover, dementia prevalence increases sharply with age, doubling every 5 years beyond 65 years (Hugo & Ganguli, 2014). Dysphagia is particularly common in dementia, with severe Alzheimer’s disease cases showing prevalence rates of 84% – 93% (Mira et al., 2022). Reflecting this, participants in the present study also identified dementia as a leading cause of dysphagia, especially in the preparatory and oral phases. As one respondent noted: ‘At times patients with dementia come for rehabilitation. Usually, the reason behind it is their forgetfulness and not physiological’. With regard to the second theme, current trends in interventional strategies, most participants expressed interest in electrical stimulation as a potential option, as reflected in the statement: ‘Electrical stimulation is an option, which we are considering using’. At present, VitalStim therapy is rarely employed in hospitals across Pakistan, largely due to barriers such as scepticism regarding its benefits and limited training opportunities (Farooq et al., 2023). Nevertheless, evidence demonstrates that neuromuscular electrical stimulation is an effective intervention for the rehabilitation of dysphagia following stroke (Jamil et al., 2024). In the current study, participants acknowledged awareness of this technique, noting: ‘Have also read about electrical stimulation and its usefulness, however not yet used’. This contrasts with the literature, which indicates that neuromuscular electrical stimulation, when used as an adjunct to traditional therapy, can provide significant relief for tube-fed dysphagia patients (Scarponi et al., 2015) and may also be beneficial in paediatric cases (Budkowska & Kolanowski, 2025). These findings highlight a gap between evidence-based practice and clinical application in Pakistan, underscoring the need for greater training, awareness, and institutional support to integrate electrical stimulation into routine dysphagia management. In the current study, the third theme which emerged among the management options was traditional strategies, with the subthemes of postural management and manoeuvres topping the list as depicted by the statement ‘In clinical practice we work on their postural management, guide about swallowing manoeuvre, and with that diet modification. While working directly with patient and attendants involved indirectly’. This follows literature, which indicates that postural changes can help dysphagia patients move food boluses safely (Alghadir et al., 2017). Literature also indicates that postural management, along with electroacupuncture and traditional therapy, improves dysphagia (Wu et al., 2024). However, this is not the case in Pakistan, as stated by a participant ‘In clinical practice we use different manoeuvres with a diet modification and postural management to treat the patient mostly’ where neuromuscular electrical stimulation is rarely being used, however the therapists are ambitious is using this technique as stated by participants ‘Have also read about electrical stimulation and its usefulness, however not yet used’. Another subtheme, endoscopic intervention, came up; however, as stated, ‘We refer our patients for any endoscopic intervention to ENT specialists’. SLPs in Pakistan usually refer cases to Ear, Nose, Throat (ENT) surgeons, and only very few SLPs do Fibre-optic Endoscopic Evaluation of Swallowing ‘FEES’ by themselves. This is in contrast to American Speech-Language-Hearing Association (ASHA) guidelines, where SLPs with ample training and competence are considered qualified to perform FEES (Flexible Endoscopic Evaluation of Swallowing, 2025). Another theme that emerged was awareness campaigns for training. Participants indicated that ‘Trainings or refresher courses for SLPs are very important’. This is quite evident as very few SLPs are doing endoscopic evaluations due to a lack of training. Hence, refresher courses are no doubt useful to add the missing skills and knowledge in professionals (Schwid et al., 2019). Similarly, awareness campaigns for family and caregivers were another subtheme as indicated by the participants ‘Definitely, family members, attendants, people who are feeding the patient need to be trained because they play a very important role and this is possible with awareness programs’. There is no doubt that better awareness of caregivers is important to ensure proper patient care, avoid complications and improve the quality of life (Demetriou et al., 2025). Limitation of access to services was another subtheme which was highlighted with subthemes of physical access and online access, as indicated by participants ‘In Pakistan’s far-flung areas there is dearth of professionals and hence access to rehab services is limited’. This is a problem faced in developing countries due to habitation in far-flung regions, a shortage of trained SLPs, prolonged travel to receive services, and a shortage of transport, in addition to economic issues (O’Callaghan et al., 2005). Though online services were provided frequently during the coronavirus disease 2019 (COVID-19) period, participants indicated:

‘No, online sessions are very difficult as assessing muscles strength is not possible. SLP can guide online but assessment should not be online. Management may be online by directing the caregivers.’ (P2, 50 years old, Male; P14, 30 years old, Male)

Though the number of SLPs opting for online therapy is multiplying, literature also reveals barriers in implementing online services, including failure of technology. Hence, it is recommended to assess each individual case before deciding on physical or online sessions (Anghel, 2021). Hence, the current study illuminates SLPs’ perspectives on dysphagia management, revealing how systemic constraints and professional identities intersect to shape clinical practice. Rather than presenting dysphagia care as a purely technical skill, the findings highlight its socially situated nature, where workload pressures, limited standardised training, and institutional contexts converge to influence decision-making. These insights extend regional surveys by adding depth to our understanding of preferred intervention approaches, while also challenging assumptions about uniformity in practice. Importantly, the study underscores that clinicians’ interpretations of dysphagia care are embedded within broader healthcare structures, linking individual experiences to systemic debates on training and resource allocation. Although purposive sampling and reliance on a self-developed tool may limit transferability, the reflexive stance adopted and transparency in reporting strengthen credibility. By situating these findings within ongoing calls for national guidelines and professional development, the study contributes to a growing body of work advocating systemic reforms alongside individual clinician training. In doing so, it not only addresses immediate practice concerns but also invites readers to reflect on the wider implications for dysphagia care in the Indo-Pak context.

### Limitations, recommendations, and future directions

The research addressed unmet needs in the prevailing scenario in clinical scenario in developing countries like Pakistan.

This study has several limitations. Firstly, the sample size was limited, with purposive sampling focused on urban, hospital-based SLPs, which may have introduced sampling bias and recall bias and excluded rural practitioners as well as those in administrative roles. Secondly, data were collected using a self-developed, non-standardised tool. Thirdly, issues of trustworthiness arise, as the absence of member checking and formal second-coder review may affect the credibility and dependability of findings. Fourthly, the researchers’ professional background as SLPs and educators within the Pakistani healthcare context may have influenced both data collection and interpretation. Finally, although reflexive journaling and transparency measures were employed, the potential for reflexive bias cannot be fully eliminated, entailing limitations in the generalisability and reproducibility of the results. It is recommended that caution be exercised while interpreting the findings emerging from the present effort. It may be worthwhile to develop, validate, and standardise a tool for reporting and data collection for the respective audience of speech professionals serving in a typical Pakistani community. Further to this, additional, more specific tools for needs assessment may also be developed and validated to foster rigorous and comparable research with larger groups of professionals, patients, and informal caregivers. In doing so, some cultural variables must also be taken into consideration, as Pakistani society comprises multiple cultures and lifestyles. The findings of the study indicate reliance on more traditional and outdated intervention protocols that may need to be considered for revision. Digitalisation, manipulation, and direct methods for improving physiological aspects need to be investigated. Further, dysphagia clinics may be scrutinised for necessary equipment in the form of technology and skills to ascertain the efficacy of treatment plans.

## Conclusion

In conclusion, it can be divulged that although services for individuals with dysphagia are available, they remain limited and are concentrated primarily in major urban centres. The service delivery models are not standardised, as the research indicates. The interventions and therapies still rely on traditional supports and need to be made robust and evidence-based. There is a dearth of technological reliance; thus, standardised tools and equipment are strongly mandated. The study concludes that there is a higher workload of SLPs, which may be because of shortages of professionals, increased incidence of aetiological factors leading to dysphagia, extended periods of treatment and poorer awareness. A multidisciplinary team approach and the need for awareness campaigns were considered essential. An extensive study is recommended to identify the existing technological support available to speech professionals in hospitals, using a larger sample. Further, the efficacy of online therapy options for dysphagic clients, especially to reach out to patients at some distance, may be explored. In summary, referring to this study, the professionals (SLPs) do not have many options available to them when it comes to managing a dysphagic client; hence, their reliance on traditional and biomedical approaches is increased. In the aftermath, they often resort to non-invasive procedures, which, although safer, tend to be less effective and more time-consuming. Further to this, constraints within the system hamper their navigational options, which seriously call for technology-based infrastructure.
